# Gynæcological and Obstetrical Society of Baltimore

**Published:** 1889-03

**Authors:** 


### THE GYNÆCOLOGICAL AND OBSTETRICAL SOCIETY OF BALTIMORE.

*Stated Meeting held January 8, 1889.*

The President, Dr. Thos. Opie, in the Chair.

Dr. Wm. E. Moseley reported a case of

### SUPPURATING PAR-OVARIAN CYST.

Had this been a case of simple par-ovarian cyst, I should not have considered it worthy of a special report, but in general particulars the case is exceptional. In the first place it occurred in a mulatto woman, such growths being extremely rare in the colored race. Secondly, it was a *suppurating* par-ovarian cyst, and thirdly it had ulcerated through into the left Fallopian tube and so discharged some of its contents through the uterus. Mrs. T., a mulatto woman, presented herself at my private dispensary on the third of last October (1888). She was about thirty-five years of age, had been married seventeen years, but had never been pregnant. Up to last July her menstrual history had been perfectly normal. Three or four years before, she first noticed a slight tumefaction of her abdomen, which increased and diminished by turn, but gave her no particular discomfort, and apparently had no marked effect upon her general health. In July, 1888, her menstrual flow appeared as usual, but did not again appear up to the time of her consulting me. Co-incident with the cessation of her menstrual flow the tumor in her abdomen began increasing in size, and her general strength failed.

Examination showed the following condition: The abdomen contained a tense, perfectly smooth, elastic tumor, reaching from the pubis to about half way between the umbilicus and ensiform cartilage. It was uniformly flat on percussion except in either flank. Fluctuation was very marked in all directions, and the abdominal wall seemed to be freely movable over the surface of the growth. Changes of position made no change in the above signs. Abdominal measurements at this time were as follows: Circumference at umbilicus $30\frac{3}{4}$ inches, pubis to umbilicus $6\frac{1}{2}$ inches and ensiform cartilage to umbilicus $8\frac{1}{2}$ inches. Digital examination showed apparently a small uterus, which was flattened up against the pubis, with no other sign of uterine disease. Fluctuation could not be made out in the vagina. The woman's general condition was bad. She was very anæmic and weak, appetite poor and bowels constipated. I determined to watch her for a time, to build her up as much as possible, and to act in accordance with future developments, feeling certain that she could not well be in a worse condition for operation, and could probably be gotten into a better one.

She had suffered much from malarial troubles, and was put upon arsenic, iron and quinine, with good food and rest in bed. Her abdominal measurements during the following two months changed but very slightly. Her temperature varied between 100° and 102° in the axilla, and her pulse ranged from 90 to 100 beats per minute. But her general condition did improve very markedly.

On November 13th, I measured the depth of her uterine canal, and found it

2¼ inches. At no time was there any cough or lung symptom. Taking all the facts into consideration, I felt that the most probable diagnosis was a tense, rather thick-walled monolocular cyst of the left ovary, and Dr. T. A. Ashby, who kindly saw her in consultation with me soon after this, reached the same result. The persistent high temperature, admitting that the diagnosis was correct, of course pointed in the direction of suppuration.

On December 5th, after the abdominal walls had been scrubbed with 1-2000 solution of bichloride of mercury, with the patient under ether, administered by Dr. Keyser and assisted by Drs. Ashby, Platt and Robert Johnston, I made the usual incision, a trifle less than two inches in length. No abdominal adhesions were found. Upon puncturing the cyst, a free flow of foul-smelling pus occurred and a little over a gallon was drawn off. Although some flowed down over the abdominal wound, this was kept so tightly closed by tension upon the sack, that I am satisfied none entered the abdominal cavity. There was a considerable adhesion to the omentum, which was ligated and cut. Great difficulty was found in separating the tumor from the uterine adhesions, which were unusually firm and extended well over to the right broad ligament and tube. All adhesions were tied with carbolized catgut and all bleeding vessels controlled in the same manner. The pedicle proved to be the whole length of the left broad ligament. This being so large and containing some large size vessels, was tied with carbolized chinese silk, the stump thoroughly seared with a Paquelin knife at a little more than a black heat and dropped back into the abdominal cavity. The abdomen was thoroughly washed out with warm boiled water, cautiously dried and the wound closed with seven carbolized silk sutures. The external dressing consisted of a piece of muslin upon which had been spread a considerable layer of antiseptic ointment (consisting of one grain of mercurial bichloride rubbed up in an ounce of benz. oxide of zinc ointment), upon this a piece of oiled silk, to prevent the gauze from coming in contact with the adhesive plaster, enough absorbent cotton, which had been freely baked, to fill up the depression between the prominent iliac bones and a cotton binder. Then the patient was put into bed, between woolen blankets, and artificial heat applied.

The time consumed was about one hour and a half, most of it having been occupied in separating the tumor from its adhesion. The patient rallied well from the ether, vomited but once and showed no sign of extreme shock.

Four hours after the operation, my patient had a temperature of 98.2° in the axilla, and a full, smooth pulse of 83. The only anodyne given was one subcutaneous injection of a quarter of a grain of morphia. She took a cup of black coffee in teaspoonful doses, and as her stomach was undisturbed by it, was ordered milk in teaspoonful doses every half hour; this was gradually increased, and at the end of forty-eight hours, she was given beef tea (home-made beef tea and a teaspoonful of beef peptonoids to each cup full). The highest temperature reached was 101.5°, on the evening of the second day. The pulse was abnormally slow and was accompanied by a rather scanty secretion of urine. Infusion of digitalis failed to cause improvement in this respect, but the flow promptly increased upon the free use of Lythia water and the pulse became normal. The bowels moved naturally on the third day, causing no pain. On the fourth day menstruation appeared, the first time for five months.

Three of the abdominal sutures were removed on the eighth day when union was perfect. The remaining sutures were removed on the fourteenth day.

On the sixteenth day the temperature suddenly went up to 102.1° in axilla, the rise evidently of malarial origin. Nine grains of quinine sulphate, in divided doses inside of six hours, reduced the temperature to normal, where it remained.

The examination of the cyst was kindly made by Prof. Wm. H. Welch, of the Johns Hopkins University, and his report was as follows:

*Examination of tumor removed by Dr. Moseley.*—The specimen is a unilocular cyst, has a glistening, grayish-white appearance, and presents numerous shreds of tissue, consisting of adherent bits of omentum and fibrous tissue. In the pedicle is included the abdominal extremity of the Fallopian tube, which measures thirteen ctm. in length. The Fallopian tube is intimately incorporated with the wall of the cyst, its fimbriated portion being obliterated. The wall of the tube is considerably thickened, its lumen dilated to about a half ctm. near the pedicle and becoming larger toward its communication with the interior of the cyst. A probe inserted into the tube passes readily into the interior of the cyst, through an opening about one ctm. in diameter, situated on the inner wall of the cyst.

Upon opening the cyst, one-half a liter of reddish gray, somewhat viscid fluid, mixed with dark red and greyish blood clots, escapes.

The diameter of the cyst is 23 ctm. The average thickness of its wall 3 m. This wall is composed of three layers, an inner of a yellowish grey, sloughy appearance; a middle of a gray, dense, fibrous aspect; and an outer corresponding to thickened peritoneum, which can be stripped off.

The inner surface of the cyst is irregular, yellowish, necrotic in appearance. There is an oval, depressed, ulcerated area, eight inches in length and five in breadth around the opening of the Fallopian tube into the cyst.

Upon microscopical examination no epithelium is found lining the cyst, but in its place a mass of pus cells, Gluge's corpuscles and fatty granules.

The fluid contained in the cyst is composed of red blood-corpuscles, pus cells, most of which contain fatty molecules, and many are converted into Gluge's cells, free, fatty granules, and granular detritus without definite character.

*Diagnosis.*—Par-ovarian cyst, which has suppurated and has ulcerated into Fallopian tube. The unilocular character of the cyst and its intra-ligamentous situation indicate that it is of par-ovarian origin. The epithelial lining has been destroyed by the suppurative process.

The communication with the Fallopian tube is undoubtedly secondary, but it is not possible to say positively whether the ulceration is from the tube into the cyst or in the reverse direction. The extent of the ulcerating cyst around the opening into the tube would suggest that the direction of the ulceration has been from the cyst into the tube.

The free communication between the interior of the cyst and the cavity of the Fallopian tube, which is patent throughout the 13 ctm. of its length removed, would make it possible for the cyst's contents to escape through the uterus and vagina. It would be interesting to learn from the clinical history whether such a discharge of the contents of the cyst ever took place.

After receiving Dr. Welch's report, I carefully questioned the patient regarding any free, purulent discharge from the vagina, and satisfied myself that such had occurred shortly prior to the operation, although nothing had been said to me regarding it at the time of its occurrence, nor was such a discharge present when I made a vaginal examination.

A word regarding the antiseptic ointment that I used in dressing the abdominal wound. I have used it for past two or three years and have put it to several severe tests and it has always answered its purpose exceedingly well.

Dr. Wm. Pawson Chunn said that in the history just presented by Dr. Moseley he was struck by the fact that amenorrhœa was present in conjunction with swelling of the abdomen. This might give rise to suspicion of pregnancy and lead to maltreatment unless the use of the sound was refrained from. If the tumor was considered to be a par-ovarian cyst, tapping might have been undertaken as has been advised by Spencer Wells, but in this case would have been probably useless, or dangerous, on account of the putrid contents of the sac. Where there is pus in a sac, tapping may let out the contents into the abdominal cavity, or may produce hemorrhage in the cyst, which danger may be avoided by an abdominal incision. The proper plan here was to extirpate the sac. He had recently seen a case in consultation which he diagnosed as a par-ovarian cyst, but which did not turn out so fortunately as Dr. Moseley's case. The patient showed on examination a spherical cyst, with flaccid wall, movable and fluctuating equally in all directions, evidently unilocular in character. Operation was delayed until the attending physician could return to town, but in the meantime the woman fell down stairs and the tumor burst. She passed great quantities of straw-colored urine, and the tumor partially disappeared, but she went on from bad to worse, and finally died, having refused operation.

Dr. Moseley, in closing, said that in the diagnosis pregnancy had been carefully eliminated. The introduction of the probe was postponed until the case had been under careful observation for nearly two months. When it was used it settled the question beyond any possible doubt. The necessity for antisepsis varies inversely as the cleanliness of the patient's surroundings When we can secure *pure* air, absolute cleanliness is all that is required, but if the surroundings are bad, antisepsis is an absolute necessity. The treatment of such a case as the one reported cannot be based upon the requirements in a case of simple par-ovarian cyst. The pedicle was broad and distinct, and moreover contained large vessels, making ligation necessary. Patients, he believed, should, whenever possible, be fed by the stomach. By waiting until the stomach seems settled and then feeling one's way carefully with small doses of milk at regular intervals, no rectal feeding will be found necessary in the majority of cases.

Dr. C. O'Donovan, Jr., reported a case of

RAPID PULSE IN A HYSTERICAL WOMAN.

On November 26th, 1887, I was called hurriedly to see Mrs. S——, white, a widow for about a year, the mother of two children, both dead now. I had known her for the past three years, and had often attended her for various slight ailments more or less complicated by nervousness, that would become sometimes slightly hysterical. About six months ago she complained very much of backache and menorrhagia, which I found by examination, was caused by a retroversion and for which I had introduced a Smith-Hodge pessary, affording her considerable relief; this she was still wearing. She had never been quite herself since the death of her husband, who had consumption, and who required a great deal of nursing, which was freely given by his wife, until his death left her but a shadow of herself, thin, pale. anæmic, constantly harassed by attacks of nervousness, varying in intensity, with each of which she would experience shortness of breath and palpitation of the heart. Her husband had kept a little tobacco shop, and just managed to make enough to keep them going, but when he died the affairs of the shop got into disorder, and financial difficulties were added to her other sources of uneasiness, until she became so despondent and nervous that she could scarcely hold a needle to sew. This chronic state would frequently culminate in paroxysms of such intensity that she would be driven from the house in spite of herself, and would feel obliged to walk and walk, for an hour or more, until she would experience some relief, when she would return home in a state of exhaustion from which she would not recover for a day or more. During several of these attacks she has walked into my office, with quickened, shallow respiration, and pulse-rate about 120, an anxious, frightened countenance; and I had usually succeeded in relieving her by giving a dose of asafœtida or valerian, or some such remedy. She had always in her house, by my direction, a number of pills containing a grain each of asafœtida and sulphate of iron, which she had been in the habit of taking whenever one of those attacks would occur, and for a week or ten days after its subsidence. These seemed to afford her considerable temporary benefit, but her nervous system had never been able to regain its normal condition, in spite of iron, quinine, strychnia and the various vegetable tonics, which she took at different times. While she continued under treatment and observation, she would get on quite well; she would develop some appetite and could control herself enough to give hope of permanent cure, but as soon as she was left to herself, all of her lately acquired stamina would disappear and she would fall back into her former condition, her hands would become tremulous, her eyes would move restlessly from object to object, her feet would be pushed forward and then drawn back, or would be crossed one over the other for half a minute and then reversed; if sitting in a chair, after a few minutes she would feel obliged to change to another, so that she seemed almost like a child with chorea, except that the movements were deliberate, and lacked that convulsive jerk so characteristic of that disease. In addition to this she would lose her appetite, becoming dyspeptic and constipated. During all this while she could sleep well, passing her only comfortable hours in bed, but spending her days in misery. Her family history, and her own personal history were both good, her mother being alive and hearty, at about sixty years, and two of her sisters whom I met subsequently were healthy women; there was no suspicion of syphilitic taint. Knowing all these facts I was prepared, when summoned on the night in question, to find her suffering from a recurrence of her nervousness, but for nothing like what I found. She lay in bed, quiet enough, apparently experiencing no pain, which supposition she subsequently confirmed, the only evidence of anything wrong being her hurried, shallow respiration and the intensely anxious expression of her countenance. I had entered the room carelessly, and began to ask her about herself before feeling her pulse, as the evening was cold and I did not care to grasp her wrist with my hand before it had become less numbed. She told me that she had arisen perfectly well, but that something had happened in the morning to precipitate one of her nervous attacks, which she had endeavored to walk off, but had failed, returning worse than when she had started out, suffering principally from shortness of breath and a sense of suffocation; this had become so bad by midday that she was obliged to go to bed, where she had since remained, but without relief.

At this stage of her story I felt her pulse, and was shocked at its extreme feebleness and rapidity. In spite of its fluttering character the impulse wave of each contraction was distinct, and no dicrotism interfered, so that I could readily count the beats, which numbered *one hundred and ninety-two* in the minute. This I verified several times, at intervals of five minutes or so, and found these numbers remarkably constant. You may judge my anxiety and fear, so natural under the circumstances. The contractions of the heart muscle seemed fairly strong, and the action of the valves was all right, but the tension in the arteries was almost nothing, so that the blood streamed away from the heart without offering that resistance so necessary to the proper action of any system of valves, and the woman seemed to be bordering upon syncope that might, at any moment, become fatal from formation of a clot. She lay with her head upon a very soft pillow, so that it was not at all elevated, and had been taking, during the few hours just passed, a weak mixture of brandy and water. While this may have prevented a worse condition of things, it had not much improved them, and it required a very few seconds for me to write for a solution, containing in each half teaspoonful a grain of carbonate of ammonia and five drops of tincture of digitalis, for which I sent at once and anxiously awaited. In my endeavor not to betray my intense anxiety, I overdid myself, and my patient, who had been lying easy and totally unconscious of anything very unusual in her condition, seeing me evidently worried, and, worse than all, trying to dissemble, began to be frightened and uneasy herself, so, more to quiet her than for any other purpose, and to gain time, I placed my thermometer under her tongue very carefully, giving her minute directions about keeping her mouth closed, and ordering her to be sure to retain it beneath her tongue until I removed it. This simple maneuver had the desired effect, and kept her quiet until the medicine arrived. There was not the slightest elevation of temperature. I administered at once ten drops of tincture of digitalis and two grains of carbonate of ammonia, and experienced considerable relief when I found that her pulse began to improve in tone and gradually decreased in frequency; her respirations, at the same time, grew deeper and more satisfactory. After about half an hour I administered half a teaspoonful of the same mixture, affording her much relief. Finding that she continued to improve I left her then, but ordered that the mixture be continued each hour in half teaspoonful doses until she fell asleep.

November 27th. When called the next morning I found her in bed still, but feeling very much more comfortable, although she was worn out and prostrated after the experience of yesterday. Her pulse had fallen to 132, but was much fuller, and felt satisfactory. After I left her she took five doses of the medicine, all before 2 A.M., about which time she fell asleep, and after waking she felt that she did not require any more. She told me that she had once before had a similar attack, and had been attended by a physician hastily summoned in the neighborhood, who seemed, she said, even more scared than I had shown myself, to which I could only reply, that I could well believe it.

November 28th. When I called the next day, she met me in the store, feeling perfectly well. Her pulse was then 72, full, strong and regular.

Dr. F. E. Chatard, Jr., related some particulars of a case of rapid heart in a young hysterical woman, in which the rapidity of pulsation varied from 150 to 170 during a period of four weeks. The patient at the same time was suffering from hysterical aphonia and other marked phenomena of similar origin. There was no rise of temperature and no pulmonary or cardiac lesion to account for the condition. As she returned to a more normal and healthy state of her nervous system, it was found that there was a reduplication of each heartbeat, and each alternate beat became gradually weaker and weaker, and was ultimately eliminated, leaving her normal pulse about 75.

Dr. Wm. E. Moseley related the case of a lad in the Boston House of Correction, whose pulse became very rapid, about 160, soon after being placed in a *dark* cell. The rapidity of the pulse was apparently due entirely to the prisoner's nervous condition, and subsided promptly upon his being taken out of solitary confinement. Somewhat akin to this class of cases, was that of a lady, who two or three days after an abortion, had a *nervous chill*, during which the temperature reached over 107° F., and the pulse was correspondingly rapid. In the course of an hour pulse and temperature were practically normal.

Dr. Geo. W. Miltenberger said: One of the most remarkable and interesting cases bearing upon unusual rapidity of pulse which I have ever seen, I met with a few months since. On September 21st last I was called in consultation by my friend Dr. T., to see Mr. H., with him and another consultant, one of our most distinguished physicians, who had already seen him. Interested in the history of his case I spent at my first visit an hour in the examination. I found a man of about 56 years of age, large, robust, athletic, who, I was told, had been noted for his physical powers throughout his life. Many years of his life had been passed in mechanical pursuits, requiring great muscular strength, as well as endurance. There was no history of constitutional taint, hereditary or acquired. He had been addicted to no vices, had not used liquor to any extent but had used tobacco, both smoking and chewing very freely. There was no albuminuria, there was no organic disease of the heart and lungs, there was no impairment of sensation or motion, and no mental affection whatever. For twenty years he had attacks of cardiac palpitation and irregularity at intervals, which were pretty promptly relieved by cardiac stimulants and nervines, and did not interfere with his usual avocations. Some two or three months before my visit, these attacks became aggravated and so repeated as to invalid him, and force him to withdraw from business. And now for the first time he had with them excessive and distressing dyspnœa. At a still later period, some weeks before I saw him, the stomach became affected and he had with his paroxysms, nausea and occasional vomiting.

The attacks coming on every day or two, would now continue for several hours, and resisted every means for his relief, or the relief would be only partial and temporary. All the nervines, sedatives, antispasmodics and heart tonics were tried but in vain. Nitro-glycerine was tried but without avail, nitrite of amyl at first seemed to afford a little relief but was afterwards useless. First heart, second lungs, third stomach, followed in sequence in this history. There being no organic disease of heart, lungs, stomach, liver or kidney, and the peripheral distributions of the pneumogastric all participating in regular sequence, the question of necessity arose was there any organic lesion central at its point of origin or along the course of its trunk. There was no alteration of sensation or motion, there was no mental aberration, there was no syphilis, there certainly was no neoplasms, which would have gone on for twenty years in this slow course without other special indications or results, there was no scrofulous tendency and no enlarged glands, along the course of the nerve. There was no evidence whatsoever of any intrathoracle or tumor or glands could produce such effect by pressure. I was forced to the conclusion that it was purély functional, affecting first the cardiac distribution, secondly the pulmonary and thirdly the gastric. The only cause to which I could attribute it was probably to the effect of excessive muscular effort in early and adult life, and the excessive use of tobacco, nicotine poisoning.

I had never seen such excessive results of tobacco. While he had used none, for over two months, and despite the most careful and untiring treatment, his troubles had been exaggerated instead of being ameliorated. This was the result of my first visit, during which he was perfectly comfortable, pulse about 70 or 80, respiration normal, stomach quiet; he was cheerful, talked freely without distress, and was comparatively a well man. The next morning I accidentally met Dr. Tiffany on the street, who requested me to go at once to see his patient, who was then suffering an attack. I found him sitting on the side of his bed, with his head supported on the back of a chair and had been thus for hours. His dyspnœa was excessive and most distressing, face pallid, pulse 200, not counted at the wrist, but with the ear close to the heart. With regard to this, there could be no mistake, as all three of us counted them in succession. There was persistent nausea and slight mucous vomiting. The following morning his pulse was 80, respiration normal, and his whole condition altered. And so, with these alternations, he continued as long as I saw him, and as far as I know, up to his death, which occurred some weeks after my last visit.

Dr. Thomas Opie exhibited a patient with

FUNGOID GROWTH ON THE LEFT BREAST.

Mrs. F., forty-five years of age, married at twenty, and has had eleven full-term children and two abortions, her last pregnancy having ended four years ago. Her general health is poor and mental condition very much depressed. The growth began as a small nodule sixteen years ago, below the left nipple. She nursed her last child two years. A short time after weaning it, the tumor commenced to grow. On April 16th, 1888, Dr. A. L. Wolf, of Page County, Va., removed it when the size of a goose egg. Dr. Wolf writes that "after making the incision the tumor was readily enucleated by his knife handle and finger. The wound healed by the first intention and left only a slight scar. Four months afterwards, a second tumor came, about two inches from the seat of the first one, and in six weeks attained to a growth which weighed four pounds. The mass removed embraced the whole gland. The incision again healed by first intention, but the scar did not become so pale and small as did the other. About six weeks after the second operation there arose a sleek, purplish-red projection, which was followed by others, which soon bursted through the skin, with the present result: medullary sarcoma or fungus hæmatodes."

Mrs. F. was admitted into the hospital December 11th. The tumor, the odor of which was unbearable, was dressed twice a day with surgeon's wool, after washing with a solution of permanganate of potash ʒj to Oj.

The fungoid growth was removed December 18th. An attempt was made to effect it with the thermo-cautery, but it was quickly decided, in view of the hæmorrhage, to circumscribe and rapidly excise it, with the scalpel. An incision through the sound skin, an inch outside the diseased tissues, was made. The whole of the pectoralis major muscle under the mass was removed. The arteries were caught up as soon as severed, with catch forceps. The wound left, measured nine inches from above downwards and seven from side to side. During the latter part of the operation, the patient sank from loss of blood or shock and was given hypoderms of whisky. She rallied satisfactorily, the wound was dressed and she was put to bed. The pulse rate and temperature from the day after the operation to the time of her death on the tenth day, were as follows:

| | A.M. | | PM. | |
|---|---|---|---|---|
| | Pulse. | Temp. | Pulse. | Temp. |
| Dec. 19th, 1888. | 110 | 100 | 112 | 100.4 |
| 20th, " | 116 | 100.2 | 116 | 100.2 |
| 21st, " | 108 | 99.5 | 116 | 100.2 |
| 22d, " | 116 | 99.4 | 130 | 100.5 |
| 23d, " | 137 | 99.4 | 140 | 100 |
| 24th, " | 128 | 98.5 | 140 | 99.8 |
| 25th, " | 130 | 99.5 | 128 | 100 |
| 26th, " | 130 | 103 | 140 | 103 |

On the second day after the operation the patient became very delirious, and continued in an insane condition until death ensued.

Billroth in his recent work on "Diseases of the Mammary Gland," page 63, "Wood's Library," 1888, says the four cases reported (by him) show how little infectious soft sarcoma of the mamma depends on age. The cases were in the 14th, 31st, 42d and 65th years. He says the differentiation of soft sarcoma from soft carcinoma and the different forms of soft sarcoma from one another, is principally and in part exclusively based on exact microscopic examination and is the result of the progress made in the last ten years. He calls attention to errors of Velpeau, Erichsen, Gross and others in classifying such cases as these in the category of "encephaloids."

The following report was made by Dr. Keirle after examination of a specimen of the growth under the microscope: "The tumor is a recurrence after removal; in gross it looks like white brain matter (medullary encephaloid), with numerous fine red streaks; in consistence it is so soft as to easily break up on handling. Microscopically, it is made up of medium-sized spindle cells, with homogeneous, gelatinous intercellular substance. Much of this structure is undergoing fatty degeneration; the mammary tubes are full of proliferating large roundish cells, so that a lumen cannot be discovered. On cross section these tubes somewhat resemble epithelial nests (perlkrebs, globes epidermiques): scattered throughout the section are numerous large, round cells with large nuclei; also numerous vessels with spindle cell walls.

"The growth is a sarcoma with epithelial proliferation, in reference to which the following is quoted from Billroth's Surg. Syd. Soc. Ed., vol. ii, p. 426: 'There is reason to fear general infection, and besides this, a transformation into cancer in consequence of epithelial proliferation becomes possible.'"

